# Tannin Supplementation Improves Oocyte Cytoplasmic Maturation and Subsequent Embryo Development in Pigs

**DOI:** 10.3390/antiox10101594

**Published:** 2021-10-12

**Authors:** Zhi Yin, Jing-Tao Sun, Hong-Di Cui, Chao-Qian Jiang, Yu-Ting Zhang, Sanghoon Lee, Zhong-Hua Liu, Jun-Xue Jin

**Affiliations:** 1 Key Laboratory of Animal Cellular and Genetics Engineering of Heilongjiang Province, College of Life Science, Northeast Agricultural University, Harbin 150030, China; zyin@neau.edu.cn (Z.Y.); sunjt@neau.edu.cn (J.-T.S.); cuihongdi217@163.com (H.-D.C.); jiangchaoqianneau@163.com (C.-Q.J.); zhangyuting217217@163.com (Y.-T.Z.); 2 Laboratory of Theriogenology, College of Veterinary Medicine, Chungnam National University, Daejeon 34134, Korea; sanghoon@cnu.ac.kr

**Keywords:** tannins, cumulus expansion, cytoplasmic maturation, embryo development, MPF

## Abstract

To investigate the effects of tannins (TA) on porcine oocyte *in vitro* maturation (IVM), different concentrations of TA (0, 1, 10 and 100 μg/mL) were supplemented with a maturation medium and the COCs and subsequent embryonic development were examined. The results showed that 10 µg/mL TA significantly improved the cumulus expansion index (CEI), cumulus-expansion-related genes (*PTGS1*, *PTGS2*, *PTX-3*, *TNFAIP6* and *HAS2*) expression and blastocyst formation rates after parthenogenetic activation (PA), *in vitro* fertilization (IVF) and somatic cell nuclear transfer (SCNT) compared to the control groups, but not oocyte nuclear maturation. Nevertheless, 10 µg/mL TA dramatically enhanced the mRNA expression of oocyte-development-related genes (*BMP15*, *GDF9*, *CDC2* and *CYCLIN B1*), GSH, ATP, SOD1, PGC1α, BMP15, GDF9 and CDC2 levels and reduced intracellular ROS level in porcine oocytes. These results indicated that porcine oocyte cytoplasmic maturation was improved by 10 µg/mL TA treatment during IVM. In contrast, a high concentration of TA (100 μg/mL) significantly decreased the CEI and *PTGS1*, *PTGS2*, *PTX-3* and *HAS2* mRNA expressions in cumulus cells, and reduced oocyte nuclear maturation and the total cell numbers/blastocyst. In general, these data showed that 10 μg/mL TA supplementation has beneficial effects on oocyte cytoplasmic maturation and subsequent embryonic development in pigs.

## 1. Introduction

Oocyte *in vitro* maturation (IVM) is one of the most critical steps in *in vitro* embryo production, which promises to improve the production of genome-modified domestic animals [1]. In addition, the porcine zygote metabolic reserves, the timing of embryo development to blastocyst, amino acid metabolism, zygotic genome activation (ZGA), the genome structure and physiological characteristics are similar to humans [2]. Therefore, the porcine oocyte is an ideal model to understand the development of female germ cells in humans, and to evaluate food safety and toxicology during embryogenesis.

Tannins (commonly referred to as tannic acids, TA) are a class of polyphenolic compounds that are found in a variety of plant-based foods, especially berries, red wine, coffee, nuts and beans. TA is a powerful antioxidant, and therefore exhibits various pharmacological properties such as anti-toxic, anticancer, antiallergic, antiviral and antibacterial abilities [3,4,5,6]. It is thought that TA could protect leaves against insects by deterrence and/or toxicity and induce reactive oxygen species (ROS) development in prostate cancer cells by inhibiting lipid metabolism [3,7]. Moreover, some hydrolysable TAs have been identified as potential inhibitors of the SARS-CoV-2 virus, which causes COVID-19 [8]. It also presents in the reproductive tracts of males and females subjects [9]. In the female reproductive system, TA could effectively adjust ovarian follicles’ health and development by regulating reproductive hormones such as the follicle-stimulating hormone (FSH), progesterone (P4) and the luteinizing hormone (LH) *in vivo* [10]. In addition, the opportunity to exploit phytogenic substances e.g., tannins, has gained attention in livestock nutrition as natural feed additive alternatives for antibiotics to enhance livestock productive and reproductive performances [11]. In *in vitro* experiments, TA exerted a powerful biological effect in finely modulating capacitation and sperm fertilizing ability [12], while TA supplementation to frozen thawed boar also could improve IVF efficiency [13]. Furthermore, TA supplementation reduced ROS and enhanced the GSH level during IVM [14], and it also might decrease polyspermy *via* the inhibition of boar sperm hyaluronidase activity [15]. Nevertheless, the underlying molecular mechanisms of TA are unclear, and there is limited information regarding the effects of TA on reproduction and, especially, on female germ cell development.

To understand the function of TA on porcine oocyte maturation during IVM, we analyzed the cumulus expansion, oocyte maturation and oocyte-development-related markers involved in nuclear maturation and cytoplasmic maturation, and embryo developmental competence after parthenogenetic activation (PA), IVF and somatic cell nuclear transfer (SCNT).

## 2. Materials and Methods

### 2.1. Chemicals

All reagents and chemicals were purchased from the Sigma-Aldrich Chemical Company (St. Louis, MO, USA) unless otherwise stated.

### 2.2. In Vitro Maturation of Porcine Oocytes

Porcine ovaries were obtained at a local slaughterhouse and transported to the laboratory at 28–35 °C. The porcine cumulus–oocyte complexes (COCs) were recovered from follicles 3–6 mm in diameter by syringe aspiration with an 18-gauge needle. The COCs with a homogeneous cytoplasm of oocytes were pooled and washed 3 times with tissue culture medium-199 (TCM-199; Invitrogen, Carlsbad, CA, USA) containing 5 mM sodium hydroxide, 10 mM N-piperazine-N′-[2-ethanesulfonic acid] (HEPES), 2 mM sodium bicarbonate, 0.3% polyvinyl alcohol (PVA) and 1% Pen-Strep (Invitrogen, Carlsbad, CA, USA). Next, 50 COCs were put into a 500 μL IVM culture medium comprising TCM-199 supplemented with 0.6 mM cysteine, 0.1 mM sodium pyruvate, 10% porcine follicular fluid (*v*/*v*), 10 ng/mL epidermal growth factor (EGF), 10 IU/mL luteinizing hormone (LH), 10 IU/mL follicle-stimulating hormone (FSH) and different concentrations of TA (0, 1, 10 and 100 μg/mL; 403040, Sigma). Then, the pooled COCs were incubated at 38.5 °C under 5% CO_2_ in 95% humidified air for IVM. Following 21 h of maturation with hormones (LH and FSH), the COCs were cultured in a hormone-free IVM medium for an additional 21 h.

### 2.3. Evaluation of Cumulus Expansion Index

The cumulus expansion index (CEI) was assessed by the morphology of COCs and distinguished to five grades after 42 h IVM as described previously [16]. Briefly, no cumulus expansion was indicated as “Grade 0” (obtaining a score of 0), which was characterized by the detachment of cumulus cells from the oocyte to assume a flattened monolayer of fibroblastic appearance. A “Grade 1” indicated no expansion, with spherical cumulus cells that remained compacted around the oocyte, which obtained a “1 score”. When the cumulus cells had expanded only the outermost layers (obtaining a score of 2), this fell into “Grade 2”. A “Grade 3” indicated all cumulus cells layers prominently expanded, but not including the corona radiata (obtaining a score of 3). Lastly, “Grade 4” indicated the maximum degree of expansion including the corona radiata (obtaining a score of 4). The CEI value was calculated using the following equation: CEI = 0 (score) × Grade 0 COCs numbers + 1 (score) × Grade 1 COCs numbers + 2 (score) × Grade 2 COCs numbers + 3 (score) × Grade 3 COCs numbers + 4 (score) × Grade 4 COCs numbers/Total number of cultured COCs (Grade 0 + 1 + 2 + 3 + 4).

### 2.4. Assessment of Oocyte Nuclear Maturation

After 42 h of IVM, COCs were denuded with 0.1% hyaluronidase by pipetting and were then washed 3 times in Tyrode’s albumin lactate pyruvate (TALP) medium. The denuded oocytes were evaluated with a microscope (TE2000-S, Nikon, Tokyo, Japan) and classified as immature (without the first polar body, 1st PB) or at metaphase II (MII, with the 1st PB).

### 2.5. Detection of GSH and ROS Levels in Porcine Oocytes

To measure the intracellular GSH and ROS levels, denuded oocytes (MII stage) were sampled in Dulbecco’s phosphate-buffered saline (DPBS; Invitrogen) with added CellTracker Blue CMF2HC (4-chloromethyl-6.8-difluoro-7-hydroxycoumarin; Invitrogen) and H2DCFDA (2′,7′-dichlorodihydrofluorescein diacetate; Invitrogen). Each treatment group (using 15 oocytes for 1 replicate) was incubated (in the dark) for 30 min in 10 µM CellTracker Blue and 10 µM H2DCFDA. Then, the samples were washed 3 times with DPBS, oocytes were placed into 4 µL droplets of TALP-HEPES and fluorescence was observed under an epifluorescence microscope (TE2000-S, Nikon, Tokyo, Japan). The excitation and emission wavelengths were 371/464 nm for CMF2HC and 492~495/517~527 nm for H2DCFDA, respectively. The intensity of fluorescence was measured by Image J software.

### 2.6. Measurement of ATP Contents in Porcine Oocytes

Denuded oocytes (using 15 oocytes for 1 replicate) were washed 3 times in DPBS and fixed with 4% paraformaldehyde for 1 h, washed 3 times and incubated in DPBS supplemented with 500 nM BODIPY FL ATP (BODIPY-ATP; A12410; Molecular Probes, Eugene, OR, USA) for 1 h in the dark. Oocytes were washed 3 times in DPBS and mounted on cover slips. Images of each oocyte were captured using an epifluorescence microscope (TE2000-S, Nikon, Tokyo, Japan). The excitation and emission wavelengths were 504/514 nm for BODIPY FL ATP. The intensity of fluorescence was measured by Image J software.

### 2.7. Analysis of Protein Expression by Immunofluorescence Staining in Oocytes

Porcine MII-stage oocytes were fixed with 4% paraformaldehyde for 30 min and placed in PBS containing 1% Triton X-100 (*v*/*v*) for 30 min at room temperature. Then, nonspecific sites were blocked with 2% bovine serum albumin (BSA) in PBS overnight at 4 °C. Oocytes were incubated with a rabbit polyclonal antibody against SOD1 (ab13498, Abcam, Cambridge, England), PGC1α (ab54481), BMP15 (PA5-34401, Invitrogen, diluted 1:200), GDF9 (ab93892) or CDC2 (ab18) at 37 °C for 2 h. A goat anti-rabbit fluorescein isothiocyanate-conjugated secondary antibody (1:200; Jackson Immuno Research Laboratories Inc., West Grove, PA, USA) or a Donkey anti-mouse IgG (H+L) highly cross-adsorbed secondary antibody (1:200; Alexa Fluor 546, A10036, Invitrogen, Carlsbad, CA, USA) was applied for 2 h. In addition, oocyte nuclei were labeled with 10 µg/mL Hoechst 33342 for 10 min. Oocytes were washed 3 times in PBS and then mounted on glass slides and evaluated with the same exposure times and adjustments under an epifluorescence microscope (TE2000-S, Nikon, Tokyo, Japan). The intensities of SOD1, PGC1α, BMP15, GDF9 and CDC2 were measured by analyzing the oocyte images with Image J software (version 1.46r; National Institutes of Health, Framingham, MA, USA).

### 2.8. Parthenogenetic Activation

Denuded oocytes with 1st PB were selected and transferred into a chamber with two electrodes spaced 3.2 mm apart that was filled with activation solution (0.28 M mannitol, 0.1 mM CaCl_2_, 0.1 mM MgSO_4_ and 0.5 mM HEPES) and activated by electric stimulation with a single direct current (DC) pulse of 1.5 kV/cm for 60 µs utilizing a BXT Electro-Cell Manipulator 2001 (BXT Inc.; San Diego, CA, USA). Activated oocytes were washed and transferred into wells containing 500 µL porcine zygote medium-3 (PZM3), and cultured under a humidified atmosphere of 5% CO_2_, 5% O_2_ and 90% N_2_ at 38.5 °C for 7 days. To analyze each blastocyst’s total cell number, the blastocysts were dyed with 10 µg/mL Hoechst 33342 for 4 min.

### 2.9. In Vitro Fertilization

Thirty MII-stage oocytes were transferred into each 50 µL modified Tris-buffered medium (mTBM) droplet and equilibrated for 20 min in the incubator. Fresh boar semen was washed 3 times in DPBS with 0.1% BSA under centrifugation at 1900× *g* for 4 min, and then the sperm pellet was resuspended in 1 mL mTBM and adjusted to the optimal concentration with additional mTBM. Finally, 50 µL of the final sperm dilution was added to the 50 µL mTBM droplet containing the oocytes to a final concentration of 1.0 × 10^6^ spermatozoa mL^−1^ and the samples were incubated for 5 h in 5% CO_2_ in air at 38.5 °C. After IVF, the presumptive zygotes were washed three times and incubated in PZM3 in 5% CO_2_ in air at 38.5 °C for 7 days. To analyze each blastocyst’s total cell number, the blastocysts were dyed with 10 µg/mL Hoechst 33342 for 4 min.

### 2.10. Somatic Cell Nuclear Transfer

Adult pig ear tissues were chopped into small pieces and incubated in an atmosphere of 5% CO_2_ at 38 °C in Dulbecco’s modified Eagle’s medium (DMEM; Gibco) containing 20% fetal bovine serum (FBS; Gibco) (*v*/*v*) and 100 IU/mL each of penicillin and streptomycin. Oocyte enucleation was performed by an aspiration pipette to remove the first polar body and the adjacent cytoplasm in the TALP medium containing 5 µg/mL cytochalasin B. Then, using an injection pipette, we injected a donor cell into the perivitelline space of each enucleated oocyte, and the couplets were equilibrated in a fusion solution (0.28 M mannitol, 0.5 mM HEPES and 0.1 mM MgSO_4_), and transferred into a 20 µL droplet of fusion solution and exposed to a single DC pulse of 1.2 kV/cm for 30 µs using an electrical pulsing machine (LF101; Nepa Gene, Chiba, Japan). The reconstructed couplets were equilibrated in an activation solution after incubating in the PZM3 medium for 1 h, and then lined up in an activation chamber filled with activation medium and activated with a single DC pulse of 1.5 kV/cm for 30 µs using a BTX ElectroCell Manipulator 2001. SCNT embryos were washed and placed in the PZM3 medium at 38.5 °C in a humidified atmosphere of 5% CO_2_, 5% O_2_ and 90% N_2_ for 7 days. To analyze each blastocyst’s total cell number, the blastocysts were dyed with 10 µg/mL Hoechst 33342 for 4 min.

### 2.11. Analysis of Gene Expression by Quantitative Real-Time PCR

For the analysis of gene expression, total mRNAs were separately extracted from oocytes and cumulus cells for each group using the TRIzol reagent (Invitrogen, Carlsbad, CA, USA), according to the manufacturer’s protocol, and the total mRNA concentration was quantified using a NanoDrop 2000 Spectrophotometer (Thermo Fisher Scientific, Wilmington, DE, USA). Following this, complementary DNA (cDNA) was produced using amfiRivert cDNA Synthesis Platinum Master Mix (GenDEPOT, Barker, TX, USA). A PCR plate (Micro-Amp Optical 96-Well Reaction Plate, Singapore) was made by adding 1 µL cDNA, 0.4 µL (10 pmol/µL) forward primer, 0.4 µL (10 pmol/µL) reverse primer, 10 µL SYBR Premix Ex Taq (TaKaRa, Otsu, Japan) and 8.2 µL Nuclease-free water (NFW; Ambion, Austin, TX, USA), and then amplified on a StepOneTM Real-Time PCR System (Applied Biosystems, Waltham, MA, USA). The amplification protocol included an initial denaturation step for 10 min at 95 °C followed by 40 cycles consisting of denaturation for 15 s at 95 °C, annealing for 1 min at 60 °C and extension for 1 min at 72 °C. The primers used for the real-time PCR are listed in Table 1. The expression of each target gene was quantified relative to the reference gene *GAPDH* (for cumulus cells) or *RN18S* (for oocytes) using the equation R = 2^−ΔΔCt^. For ease of comparison, the average expression level of each gene from the control group was set as 1. Expression values were normalized to those of *GAPDH* or *RN18S*.

### 2.12. Statistical Analysis

The data were analyzed by one-way ANOVA followed by Tukey’s test using SPSS 17.0 statistical software (SPSS, Inc., Chicago, IL, USA). The SCNT embryo development was compared by Student’s *t*-test. A *p*-value < 0.05 was considered statistically significant. Each experiment was repeated at least 3 times.

## 3. Results

### 3.1. TA Increased Cumulus Expansion during IVM

**Figure 1:**
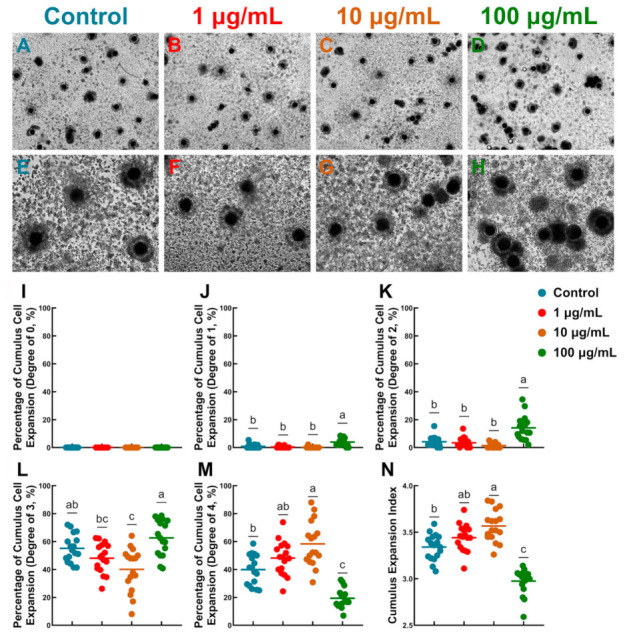
Evaluation of cumulus expansion in cumulus–oocyte complexes after 42 h of *in vitro* maturation: (**A**,**E**) Control group, (**B**,**F**) 1 μg/mL TA, (**C**,**G**) 10 μg/mL TA. (**D**,**H**) 100 μg/mL TA. (**A**–**D**) Original magnification ×40. (**E**–**H**) Original magnification ×100. (**I**–**M**) Different degrees of cumulus expansion (%); (**N**) total cumulus expansion index. Bars with different letters denote significant differences (*p* < 0.05). Results are shown as the average ± SEM of seventeen repeats of independent experiments.

A total of 3100 COCs were used in 17 replicates to evaluate whether exposure to TA at various concentrations during IVM affected cumulus expansion (Figure 1A–H). As shown in Figure 1I–N, 10 µg/mL TA significantly increased the proportion of COCs exhibiting complete cumulus expansion (CEI grade 4, 58.33 ± 3.89% vs. 39.89 ± 2.63% and 19.36 ± 1.64%, *p* < 0.05) and showed a significant decrease in the proportion of oocytes with CEI grade 3 compared to the control and 100 µg/mL TA (40.05 ± 3.73% vs. 55.10 ± 2.41% and 62.62 ± 3.09%, *p* < 0.05). Therefore, CEI was significantly increased in 10 µg/mL TA compared to the control and 100 µg/mL TA (3.57 ± 0.04% vs. 3.34 ± 0.03% and 2.97 ± 0.03%, *p* < 0.05; Figure 1N).

### 3.2. TA Affected Oocyte Maturation during IVM

**Figure 2:**
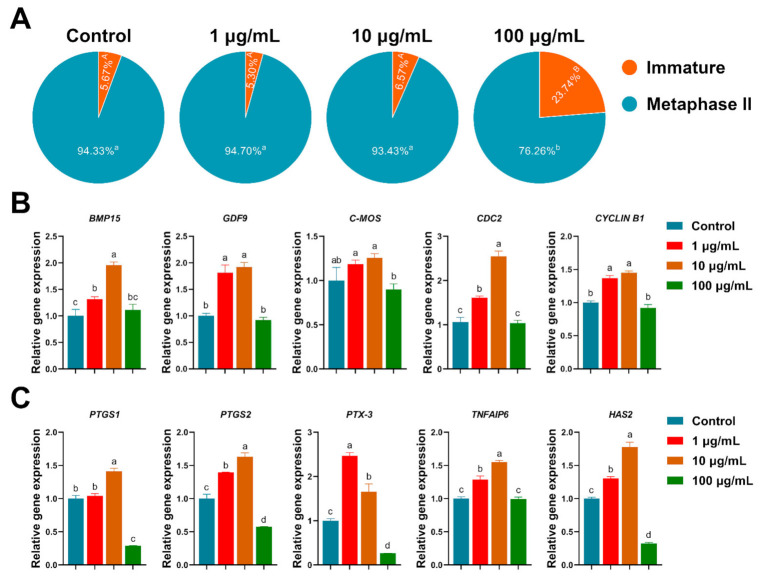
The effects of TA on the nuclear maturation of porcine oocytes and gene expression of oocytes and cumulus cells. (**A**) A total of 3239 oocytes were used in 19 independent replicates for evaluating nuclear maturation. (**B**) Oocyte developmental genes expression (GDF9, BMP15, C-MOS, CDC2 and CYCLIN B1). (**C**) Cumulus expansion genes expression (PTGS1, PTGS2, PTX-3, TNFAIP6 and HAS2). Within the same mRNA, bars with different letters denote significant differences (*p* < 0.05). Results are shown as the average ± SEM of at least three repeats of independent experiments.

A total of 2874 COCs were used in 19 replicates to evaluate the effects of exposure to TA at various concentrations during IVM upon nuclear maturation. No significant differences were observed between the control, 1 µg/mL and 10 µg/mL TA (93.03 ± 1.88%, 95.24 ± 0.74% and 93.18 ± 0.79%), but there were significant differences compared to the 100 µg/mL TA (73.59 ± 2.71%, *p* < 0.05; Figure 2A).

### 3.3. TA Regulated Cumulus Expansion and Oocyte Development Genes Expression

To detect mRNA expression, we carried out real-time PCR in cumulus cells and oocytes. Treatment with 10 µg/mL TA significantly increased the cumulus-expansion-related genes (PTGS1, PTGS2, PTX-3, TNFAIP6 and HAS2, *p* < 0.05) expression compared to the control and 100 µg/mL TA (Figure 2C). Additionally, we found that 10 µg/mL TA significantly increased the oocyte-development-related genes (BMP15, GDF9, CDC2 and CYCLIN B1, *p* < 0.05) expression compared to the control and 100 µg/mL TA (Figure 2B).

### 3.4. TA Regulated GSH, ROS and ATP Levels in Oocytes

**Figure 3:**
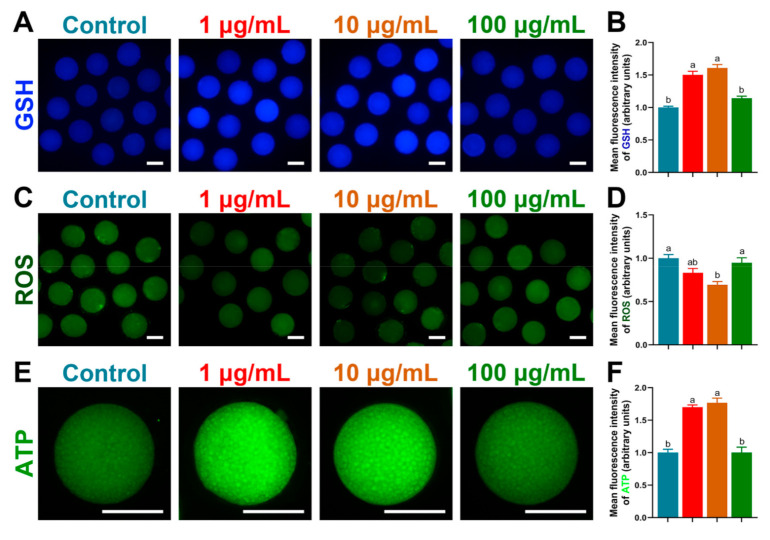
Analysis of GSH, ROS and ATP levels by fluorescence staining in porcine oocytes. (**A**,**B**) GSH levels. (**C**,**D**) ROS levels (**E**,**F**) ATP levels. Bar = 120 μm. (**B**,**D**,**F**) Bars with different letters denote significant differences (*p* < 0.05). Results are shown as the average ± SEM of at least three repeats of independent experiments.

We then evaluated the intracellular GSH, ROS and ATP levels at the MII stage of porcine oocytes (Figure 3). The levels of GSH and ATP were significantly increased in 1 µg/mL and 10 µg/mL TA compared to the control and 100 µg/mL TA (*p* < 0.05), and the level of ROS was significantly decreased in 10 µg/mL TA compared to the control and 100 µg/mL TA (*p* < 0.05).

### 3.5. TA Up-Regulated Oocyte-Development-Related Markers

**Figure 4:**
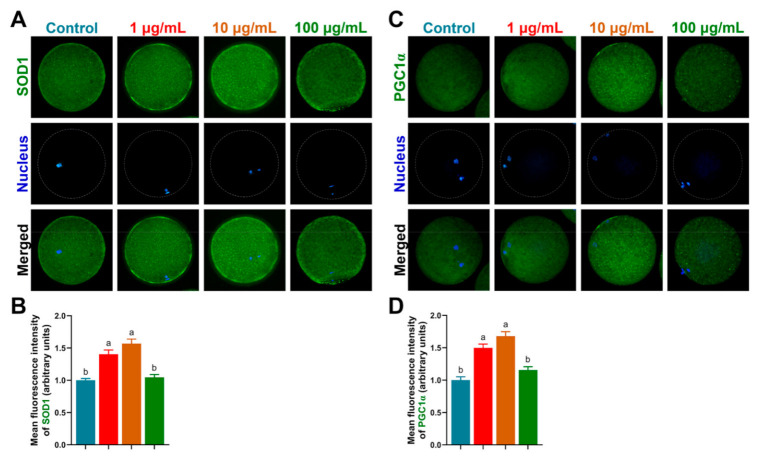
Protein levels of SOD1 and PGC1α in porcine oocytes. (**A**,**B)** SOD1 expression in oocytes. (**C**,**D**) PGC1α expression in oocytes. Bars with different letters denote significant differences (*p* < 0.05). Results are shown as the average ± SEM of at least three repeats of independent experiments.

To confirm the previous results, we analyzed the protein levels of SOD1 (anti-oxidation factor), PGC1α (mitochondrial biogenesis) and BMP15 and GDF9 (oocyte-development-related markers) in porcine oocytes. In line with the GSH and ROS results, SOD1 expression was significantly increased in 1 µg/mL and 10 µg/mL TA compared to the control and 100 µg/mL TA (1.40 ± 0.06 and 1.57 ± 0.07 vs. 1.00 ± 0.03 and 1.05 ± 0.04, *p* < 0.05; Figure 4A,B). Moreover, PGC1α was also increased in 1 µg/mL and 10 µg/mL TA compared to the control and 100 µg/mL TA (1.50 ± 0.06 and 1.68 ± 0.07 vs. 1.00 ± 0.05 and 1.16 ± 0.05, *p* < 0.05; Figure 4C,D).

**Figure 5:**
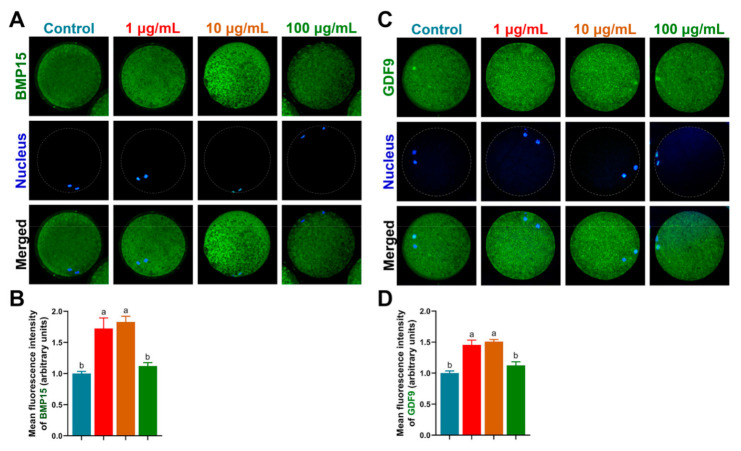
Detection of BMP15 and GDF9 protein levels by immunofluorescence in porcine oocytes. (**A**,**B**) BMP15 expression in oocytes. (**C**,**D**) GDF9 expression in oocytes. Bars with different letters denote significant differences (*p* < 0.05). Results are shown as the average ± SEM of at least three repeats of independent experiments.

**Figure 6:**
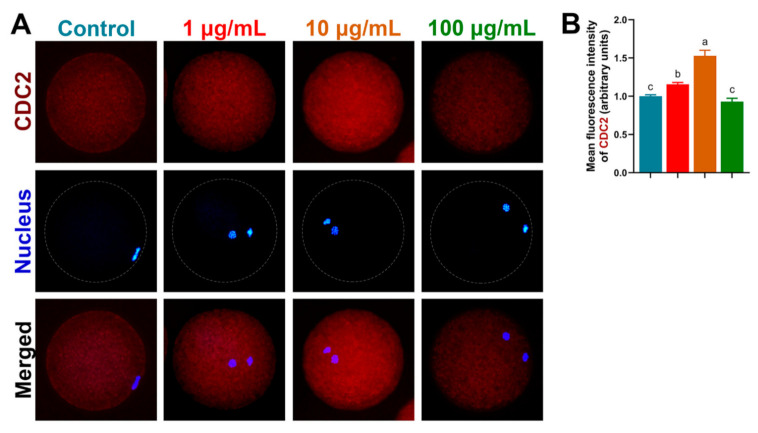
Expression of CDC2 protein detected by immunofluorescence (**A**,**B**). Bars with different letters denote significant differences (*p* < 0.05). Results are shown as the average ± SEM of at least three repeats of independent experiments.

Both BMP15 and GDF9 protein expressions (Figure 5) were significantly increased by 1 µg/mL and 10 µg/mL TA treatment compared to the control and 100 µg/mL TA (BMP15, 1.72 ± 0.17 and 1.83 ± 0.09 vs. 1.00 ± 0.03 and 1.12 ± 0.06; GDF9, 1.46 ± 0.08 and 1.51 ± 0.03 vs. 1.00 ± 0.03 and 1.31 ± 0.04, *p* < 0.05), supported by mRNA expression of *BMP15* and *GDF9*. In addition, we detected CDC2 (maturation-promoting factor, MPF) expression by immunofluorescence and found that it exhibited the highest level in 10 µg/mL TA compared to the other groups (*p* < 0.05; Figure 6A,B).

### 3.6. TA Improved Embryo Development after PA, IVF and SCNT

**Figure 7:**
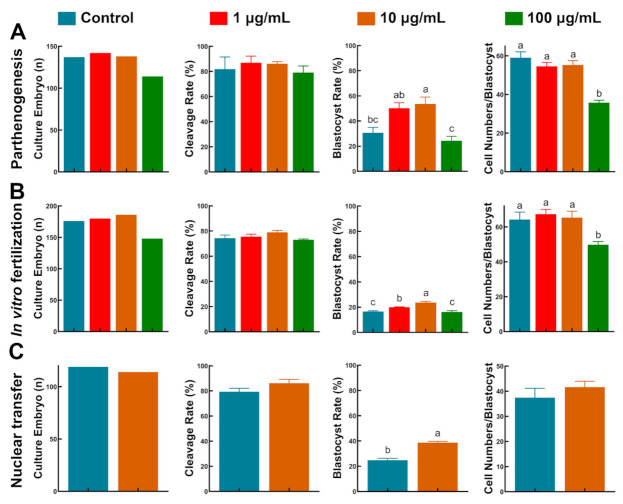
The effect of TA supplementation during *in vitro* maturation on embryonic development after (**A**) parthenogenesis, (**B**) *in vitro* fertilization and (**C**) somatic cell nuclear transfer. Bars with different letters denote significant differences (*p* < 0.05). Results are shown as the average ± SEM of at least three repeats of independent experiments.

To evaluate the oocyte developmental potential, we used 521, 690 and 233 matured oocytes to perform PA, IVF and SCNT, respectively. Embryos derived from PA and IVF had a similar developmental pattern: Although there was no significant difference in cleavage rates among the groups, the blastocyst formation rates were significantly increased in 10 µg/mL TA compared to the control and 100 µg/mL TA (PA, 53.63 ± 5.44% vs. 30.62 ± 4.31% and 24.26 ± 3.67%; IVF, 23.74 ± 0.92% vs. 16.50 ± 0.57% and 16.35 ± 0.81%, *p* < 0.05; Figure 7A,B). We then compared the SCNT embryo developmental competence between the control and 10 µg/mL TA. In line with PA- and IVF-derived embryo development, SCNT-derived embryos were not significantly different in terms of the cleavage rate and total cell number, but had significantly improved blastocyst formation rates (24.71 ± 1.68 vs. 38.69 ± 1.06, *p* < 0.05; Figure 7C).

## 4. Discussion

In recent years, great interest has been focused on using natural antioxidants in food products. These natural antioxidant molecules are derived from coffee leaves, sweet potato, ginkgo biloba leaves and tomato pulp, such as carotenoids, anthocyanins, flavanols and lycopene, respectively [17]. Numerous studies have investigated the beneficial effects of plant-derived antioxidants on the development of oocytes in various mammalian species [18,19,20,21]. Nevertheless, there is limited information regarding the effects of TA on porcine oocyte development. In the current study, our results demonstrated that supplementation of an IVM medium with 10 µg/mL TA significantly improved cumulus expansion and embryo development to the blastocyst stage following PA, IVF and SCNT when compared to the control group. Although there was no significant effect on nuclear maturation, the oocyte-development-related indicators (such as GSH, ROS, ATP, SOD1, PGC1α, BMP15, GDF9 and CDC2) were dramatically increased by 10 µg/mL TA treatment. Furthermore, 10 µg/mL TA significantly improved the expression of cumulus-expansion-related and oocyte-development-related genes.

It is well known that the cumulus cells surrounding the oocyte regulate its growth and meiotic maturation, with cell-to-cell communication *via* gap junctions [22]. The cumulus cells play essential roles in protecting the oocytes from oxidative stress [23] and saturated fatty acid stress [24,25]. Therefore, the developmental potential of the oocyte has been shown to be reflected in cumulus expansion [26]. The oocytes without cumulus cells have a much lower ratio of *in vitro* maturation and their subsequent embryo development [27,28]. It is possible that the absence of cumulus cells disrupts the metabolic balance in oocytes [29]. Moreover, our previous results demonstrated that there is higher nuclear and cytoplasmic maturation in fully expanded cumulus cells compared to the incompletely expanded COCs [1]. Therefore, the optimum expansion of the cumulus layer is necessary for the proper maturation of oocytes [30]. Additionally, higher PTGS1 and PTGS2 expression in the periconceptional cumulus is corrected with oocytes that develop into higher quality embryos [31]. HAS2 is one of the enzymes required for hyaluronan synthesis, and its expression in cumulus cells is well correlated with the cumulus expansion process [32]. Moreover, the organization of linear molecules of hyaluronan is mediated by TNFAIP6 [33] and PTX-3 [34]. It has been reported that these factors were involved in the quality of oocyte maturation [35]. In the present study, supplementation of the IVM medium with 10 µg/mL TA significantly increased the cumulus expansion of porcine COCs and the expression of genes related to cumulus expansion in cumulus cells. Based on these results, we reasonably assumed that 10 µg/mL TA treatment could promote oocyte maturation and improve oocyte quality *via* cumulus expansion.

The nuclear and cytoplasmic maturation events that occur during oogenesis up to MII confer the oocyte with developmental competence, defined as the capacity to support embryo development in all species [36]. The MAPK (mitogen-activated protein kinases) and MPF levels are believed to play a critical role during meiotic maturation [37]. In porcine oocytes, MAPK is activated at GVBD (germinal vesicle breakdown) and maintains a high level of activity until the oocyte reaches MII, when c-Mos, one of the most important maternal regulators, is activated. The MAPK activity is completely regulated after the activation of MPF *via* c-Mos, thus there is tight interaction between MAPK and MPF [38]. The activity of MPF is a critical factor in oocyte maturation. The regulation of MPF levels mainly involves the activity of Cyclin B1 and the Cdc2 kinase, which initiate oocyte meiosis to the MII stage and promote nuclear and cytoplasmic maturation [39]. Zhang and his colleague found that good-quality MII-stage porcine oocytes present a high expression of CYCLIN B1 and CDC2 [40]. GDF9 is a key mediator of cumulus expansion and induces the expression of HAS2 and PTGS2 in the presence of FSH [41]. Lin et al. indicated that GDF9 and BMP15 not only participate in cumulus expansion, but also stimulate C-MOS, CYCLIN B1 and CDC2 activity [42]. GDF9 and BMP15 proteins are localized to the oocyte cytoplasm, and strongly accumulate in the cortical region of porcine oocytes during meiotic maturation [43]. We selected BMP15, GDF9, C-MOS, CDC2 and CYCLIN B1 as maternal factors for a detailed study of protein and gene expression. In this study, despite no significant difference in nuclear maturation between groups (control vs. 1 µg/mL and 10 µg/mL TA), 1 µg/mL and 10 µg/mL TA treatment significantly increased *BMP15*, *GDF9*, *CDC2* and *CYCLIN B1* expression compared to the control group, and slightly increased *C-MOS*. Additionally, the protein expression of BMP15, GDF9 and CDC2 showed high levels in porcine oocytes, which was in line with their gene expression. These results indicated that TA could improve porcine oocyte cytoplasmic maturation by increasing BMP15, GDF9 and MPF levels.

ROS scavenging is critical for oocyte maturation and embryo development *in vitro* [44]. Generally, in a high-oxygen environment, aerobic metabolism results in the production of high levels of ROS, which in turn cause oxidative damage that affects the normal development of oocytes and embryos. GSH, the major cellular antioxidant contributing to both non-enzymatic and enzyme-dependent defense against ROS, is an inefficient substrate for SOD1-catalyzed H_2_O_2_ formation [45]. The absence of SOD1 thus leads to oxidative stress, which dramatically increases intracellular ROS levels and induces mitochondrial dysfunction [46,47]. Mitochondria exert cytoprotection through mitochondrial biogenesis, which is influenced by peroxisome proliferator-activated receptor gamma co-activator-1α (PGC1α) [48]. PGC1α has been shown to serve as an indispensable factor in the activation of the antioxidant pathway [48]. Moreover, nuclear factor erythroid-derived factor-2 (Nrf2) is a redox-sensitive transcription factor, which transcribes antioxidant enzyme synthesis such as SOD1 and GSH to provide cellular protection against oxidative damage [49]. Nrf2 signaling has been proposed to be essential for the oocyte maturation and embryo development of bovine, murine and porcine species [50]. It has been demonstrated that PGC1α could activate Nrf2 for the advertisers of mitochondrial translation factor A to prompt mitochondria function, maintenance and biogenesis [51]. Salman et al. determined that TA supplementation directly activated the PGC1α/Nrf2 pathway in traumatic brain injury models [52]. In this study, we determined the levels of several oocyte developmental markers, such as intracellular GSH, ROS, ATP, SOD1 and PGC1α, in porcine oocytes. Our data showed that all of the markers for oocyte development were dramatically changed by 10 µg/mL TA treatment. The possible reason for these findings is that the promotion of PGC1α transcription resulted in Nrf2 activation, which synthesized antioxidant enzymes SOD1 and GSH, scavenged the intracellular ROS level and facilitated mitochondrial biogenesis and bio-function, thereby improving oocyte cytoplasmic maturation by the supplementation of 10 µg/mL TA during porcine IVM.

Cytoplasmic maturation includes cytoplasmic processes that prepare the oocyte for fertilization and embryo developmental competence and occurs throughout oocyte growth and simultaneously with nuclear maturation [53]. These processes can be further divided into cytoplasmic molecular variations and organelle maturation and migration, which ultimately contribute to the resulting embryo’s developmental competence [53]. In this study, we hypothesized that the dynamic variations of cytoplasmic molecules implied that TA treatment might improve cytoplasmic maturation and subsequent embryonic developmental competence, even though there was no significant difference in oocyte nuclear maturation. In order to test our hypothesis, we carried out PA, IVF and SCNT to evaluate subsequent embryo developmental competence after TA treatment. We found that treatment with 10 µg/mL TA significantly improved blastocyst formation rates after PA, IVF and SCNT compared to the control, even though there were no significant differences in cleavage rates and total cell numbers. Interestingly, 1 µg/mL TA also significantly improved blastocyst formation after IVF compared to the control, but not after PA. This might mean that there is a smaller likelihood of polyspermy following TA treatment, due to enhanced function of the cytoplasmic organelles. Tatemoto et al. indicated that TA treatment reduced the polyspermy rate by inhibiting sperm hyaluronidase activity [15].

Surprisingly, we found that although exposure to 100 µg/mL TA significantly decreased cumulus expansion and oocyte nuclear maturation compared to the control, once these oocytes had matured, they had the same ability as the control oocytes to develop a blastocyst after PA and IVF, but there were significant differences compared to the 1 µg/mL and 10 µg/mL TA. These results agreed well with those of other authors who recorded that the polar body extrusion rate was negatively influenced in the presence of a high concentration of TA [14]. Furthermore, cyclic adenosine monophosphate (cAMP) is upstream of MAPK and controls MPF activity in the oocyte. It is synthesized in the cumulus cells and transferred into the oocytes *via* gap junctions in the COCs [54]. Generally, HAS2 expression results in the secretion of hyaluronan from cumulus cells, which is linked to the hyaluronan-associated proteins (PTX-3 and TNFAIP6) resulting in cumulus expansion [55,56]. Simultaneously, the hyaluronan binds to its receptor CD44 and induces the phosphorylation of Cx43 at tyrosine residues [57]. This process induces disruption of gap junctional communication, blocks the transport of cAMP from the cumulus cells into the oocytes and leads to MPF activation and meiotic resumption of the oocyte [57]. Our results demonstrated that the low expression of *HAS2* following 100 µg/mL TA treatment led to abnormal expression of *PTX-3* and *TNFAIP*, resulting in low cumulus expansion and incomplete inhibition of cAMP transport from the cumulus, causing the failure of the activation of MPF and MAPK in the oocyte, thereby affecting nuclear maturation. These experiments suggested the possibility of inducing, by excessive antioxidant supplementation, a dangerous condition called “antioxidant paradox” leading to “reductive stress” that in turn may impair nuclear maturation [14].

## 5. Conclusions

Our results demonstrated that 10 μg/mL of TA improved cumulus expansion, the MPF level of oocyte and subsequent embryonic development after PA, IVF and SCNT by increasing the cumulus-expansion- and oocyte-development-related gene expression, improving the level of GSH, ATP, SOD1, PGC1α, BMP15, GDF9 and CDC2, and decreasing the level of ROS. Therefore, our findings provide further insight into the function and toxicity of TA in the fields of animal reproductive physiology and human assisted reproductive technology.

## Figures and Tables

**Table 1:** Primer sequences used for real-time PCR.

Genes	Primer Sequences (5′–3′)	Product Size (bp)	Accession No.
*GAPDH*	F: GTCGGTTGTGGATCTGACCT	207	NM_001206359
	R: TTGACGAAGTGGTCGTTGAG		
*RN18S*	F: TCCAATGGATCCTCGCGGAA	149	NR_046261.1
	R: GGCTACCACATCCAAGGAAG		
*PTGS1*	F: AACACGGCACACGACTACA	121	XM_001926129
	R: CTGCTTCTTCCCTTTGGTCC		
*PTGS2*	F: ACAGGGCCATGGGGTGGACT	194	NM_214321
	R: CCACGGCAAAGCGGAGGTGT		
*PTX-3*	F: GGCCAGGGATGAATTTTAC	185	NM_001244783
	R: GCTATCCTCTCCAACAAGTGA		
*TNFAIP6*	F: AGAAGCGAAAGATGGGATGCT	106	NM_001159607
	R: CATTTGGGAAGCCTGGAGATT		
*HAS2*	F: AGTTTATGGGCAGCCAATGTAGTT	101	AB050389
	R: GCACTTGGACCGAGCTGTGT		
*BMP15*	F: CCTCCATCCTTTCCAAGTCA	112	NM_001005155
	R: GTGTAGTACCCGAGGGCAGA		
*GDF9*	F: CAGTCAGCTGAAGTGGGACA	135	AY626786
	R: TGGATGATGTTCTGCACCAT		
*C-MOS*	F: GGGAGCAACTGAACTTGGAG	115	NM_001113219
	R: AGAATGTTCGCTGGCTTCAG		
*CDC2*	F: GGGCACTCCCAATAATGAAGT	260	AB045783
	R: GTTCTTGATACAACGTGTGGGAA		
*CYCLINB1*	F: CAACTGGTTGGTGTCACTGC	126	L48205
	R: TTCCATCTGCCTGATTTGGT		

F, Forward primer; R, Reverse primer.

## Data Availability

The data are contained within the article.
